# Influence of hypercapnia and hypercapnic hypoxia on the heart rate response to apnea

**DOI:** 10.14814/phy2.16054

**Published:** 2024-06-14

**Authors:** Benjamin R. O'Croinin, Desmond A. Young, Lauren E. Maier, Sean van Diepen, Trevor A. Day, Craig D. Steinback

**Affiliations:** ^1^ Neurovascular Health Lab, Faculty of Kinesiology, Sport, and Recreation University of Alberta Edmonton Alberta Canada; ^2^ Department of Critical Care Medicine, Faculty of Medicine and Dentistry University of Alberta Edmonton Alberta Canada; ^3^ Division of Cardiology, Department of Medicine, Faculty of Medicine and Dentistry University of Alberta Edmonton Alberta Canada; ^4^ Department of Biology, Faculty of Science and Technology Mount Royal University Calgary Alberta Canada

**Keywords:** arrhythmia, bradycardia, breath‐hold, chemoreflex, diving response

## Abstract

We aimed to determine the relative contribution of hypercapnia and hypoxia to the bradycardic response to apneas. We hypothesized that apneas with hypercapnia would cause greater bradycardia than normoxia, similar to the response seen with hypoxia, and that apneas with hypercapnic hypoxia would induce greater bradycardia than hypoxia or hypercapnia alone. Twenty‐six healthy participants (12 females; 23 ± 2 years; BMI 24 ± 3 kg/m^2^) underwent three gas challenges: hypercapnia (+5 torr end tidal partial pressure of CO_2_ [P_ET_CO_2_]), hypoxia (50 torr end tidal partial pressure of O_2_ [P_ET_O_2_]), and hypercapnic hypoxia (combined hypercapnia and hypoxia), with each condition interspersed with normocapnic normoxia. Heart rate and rhythm, blood pressure, P_ET_CO_2_, P_ET_O_2_, and oxygen saturation were measured continuously. Hypercapnic hypoxic apneas induced larger bradycardia (−19 ± 16 bpm) than normocapnic normoxic apneas (−11 ± 15 bpm; *p* = 0.002), but had a comparable response to hypoxic (−19 ± 15 bpm; *p* = 0.999) and hypercapnic apneas (−14 ± 14 bpm; *p* = 0.059). Hypercapnic apneas were not different from normocapnic normoxic apneas (*p* = 0.134). After removal of the normocapnic normoxic heart rate response, the change in heart rate during hypercapnic hypoxia (−11 ± 16 bpm) was similar to the summed change during hypercapnia+hypoxia (−9 ± 10 bpm; *p* = 0.485). Only hypoxia contributed to this bradycardic response. Under apneic conditions, the cardiac response is driven by hypoxia.

## Graphical abstract

We aimed to determine the relative contribution of hypercapnia and hypoxia to apnea‐induced bradycardia. We recruited healthy males and females and measured their heart rate responses to apneas after four gas challenges: normocapnic normoxia, isooxic hypercapnia, isocapnic hypoxia, and hypercapnic hypoxia. Only hypoxia contributed to the bradycardic response. Therefore, hypoxia drives the cardiac response to apneas.

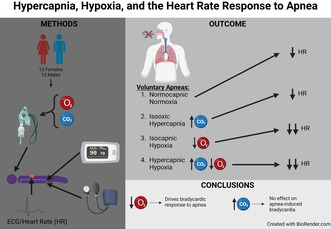

## 1 INTRODUCTION

During an apnea (i.e., breath‐hold) the human autonomic nervous system employs mechanisms to conserve oxygen including increased cardiac vagal outflow and increased peripheral sympathetic outflow (Lemaître et al., 2004; Shattock & Tipton, 2012); the former decreases heart rate and the latter induces peripheral vasoconstriction which in turn increases blood pressure. During free‐breathing the pulmonary stretch reflex inhibits cardiovagal outflow, resulting in sympathetic dominance and an increased heart rate. Only with the cessation of breathing does the silencing of the pulmonary stretch reflex permit cardiovagal outflow and subsequent bradycardia (Dampney, 2016). We recently demonstrated that the bradycardic response to apneas is greater during both acute (Busch et al., 2021) and chronic (Busch et al., 2018) hypoxia (i.e., during periods of peripheral chemoreflex activation) compared to normocapnic normoxic conditions, suggesting elevated parasympathetic signals influencing the heart under these conditions. These previous investigations were conducted during poikilocapnic hypoxia, resulting in concurrent hypocapnia. It remains unclear whether perturbations in carbon dioxide (i.e., hypercapnia) affect the bradycardia during apneas to a similar degree as hypoxia due to activation of central and (to a lesser degree) peripheral chemoreceptors.

The peripheral chemoreceptors in the carotid bodies detect changes in O_2_ and CO_2_, as well as other metabolic perturbations (e.g., temperature, pH; Guyenet & Bayliss, 2015) whereas the central chemoreceptors in the brainstem only detect changes in pH (Nattie & Li, 2012). Nonetheless, hypoxia and hypercapnia both increase heart rate and ventilation during free‐breathing conditions (Steinback et al., 2009). Despite similar autonomic responses during free‐breathing, little is known about the individual influences of hypercapnia and hypoxia compared to combined hypercapnic hypoxia on heart rate under voluntary apneic conditions. Therefore, the purpose of this study was to compare the heart rate responses to apnea during isocapnic hypoxia, isooxic hypercapnia, and combined hypoxic hypercapnic challenges. We hypothesized that apneas with hypercapnia would induce a more pronounced bradycardia than normoxia—that is, similar to what has been previously observed during hypoxia—and that apneas with hypercapnic hypoxia would induce even greater bradycardia than hypercapnia and hypoxia alone.

## 2 MATERIALS AND METHODS

### 2.1 Ethical approval

The present study was a single‐blind within‐subjects design and was approved by the University of Alberta (Pro00096895) and Mount Royal University (Protocol #103970) Health Research Ethics Boards. All participants provided written informed consent prior to testing. The study conformed with the latest version of the *Declaration of Helsinki* except for registration in a public database.

### 2.2 Study participants

Data were collected and analyzed from 26 participants (12 females; 23 ± 2 years [mean ± SD]). All participants were free of cardiovascular, respiratory, or nervous system disease as assessed by questionnaire. Additionally, no participants were taking medication, other than hormonal contraceptives (two using oral contraceptives, and five using intrauterine devices), that may impact the functioning of the cardiovascular, respiratory, or nervous systems, and all were non‐smokers. Blood pressure was within a normal range (all participants <139/85 and > 90/60 mmHg). The menstrual cycle phase and birth control medications were not controlled in females but were recorded in Table S2 (https://figshare.com/s/a3a515630a3c604ccbd7). Self‐reported race was also recorded and reported.

We calculated our sample size based on a past report employing an end‐expiratory apnea after an acute hypoxic bout (Busch et al., 2021), in which there was a large effect size (*d* = 1.0) between the heart rate responses to apnea after normoxia versus hypoxia. Based on our uncertainty about the response to a hypercapnic apnea, we chose a more conservative effect size (*d* = 0.6) for our sample size calculation. Using standard values for alpha (0.05) and beta (0.20), and a two‐tailed *t*‐test for matched pairs, we calculated our required sample size as 24. We aimed for a sex‐balanced sample, and over‐sampled males until we achieved our target of 12 females and 12 males; therefore, we ended with a final sample of 26 participants.

### 2.3 Instrumentation

Testing was conducted in a single session at the University of Alberta (676 m elevation) after participants had abstained from caffeine, alcohol, and exercise for at least 12 h. Participants laid semi‐recumbent on a bed in a temperature‐controlled room for the duration of testing (22 ± 1°C). Heart rate (HR; in beats/min) was obtained from a 3‐lead electrocardiogram (lead II) and blood pressure was recorded using finger photoplethysmography (Finometer Pro, Finapres Medical Systems). The blood pressure waveform was calibrated using manual brachial blood pressure measurements done in triplicate. A respiratory belt was used to measure changes in chest circumference (Model TN1132/ST, ADInstruments) to assist with detecting the end‐point of apneas, and peripheral oxygen saturation (SpO_2_) was measured using a pulse oximeter on the right index finger (Nellcor N‐600x, Medtronics). Participants wore a nose clip and breathed through a mouthpiece connected to a dual gas analyzer (Model ML206, ADInstruments). The mouthpiece was connected in series to a pneumotach and a two‐way non‐rebreathing valve (T‐shape series 2700, Hans Rudolph). The inspired port was connected via a hose to an automated gas control system (Airforce; Tymko et al., 2015) which allowed for dynamic control of inspired gas concentrations to reach desired end‐tidal values.

Prior to testing, all participants were instructed on how to complete an end‐expiratory apnea and were allowed to practice one apnea with and without the mouthpiece. During the protocol, a researcher assisted in removing the mouthpiece at the start of each apnea so that the resumption of breathing was done on room air. No further instructions or encouragement were given during the apneas for the duration of the protocol.

All gas conditions were achieved using a dynamic end‐tidal forcing system (Airforce) to standardize gas stimuli; this was preferable to fixed inspired gas stimuli where the ventilatory response can partially correct the resultant changes in arterial O_2_ and CO_2_. Normocapnic normoxia involved matching the end‐tidal partial pressures of O_2_ (P_ET_O_2_) and CO_2_ (P_ET_CO_2_) to those obtained in the initial baseline period (see study protocol). Hypercapnia involved an increase in P_ET_CO_2_ by +5 torr above normocapnic normoxic values. Hypoxia involved a decrease in P_ET_O_2_ to ~50 torr, targeting 80%–85% SpO_2_. Hypercapnic hypoxia involved a concurrent increase in P_ET_CO_2_ (+5 torr) and a decrease in P_ET_O_2_ (~50 torr). The end‐tidal values were chosen based on a previous investigation employing similar methods that obtained notable ventilatory and sympathetic responses (Steinback et al., 2009); the severity of the gas challenges were reduced compared to this aforementioned study during pilot testing due to challenges with excessive hyperventilation during the hypercapnic hypoxia condition.

### 2.4 Study protocol

**FIGURE 1:**
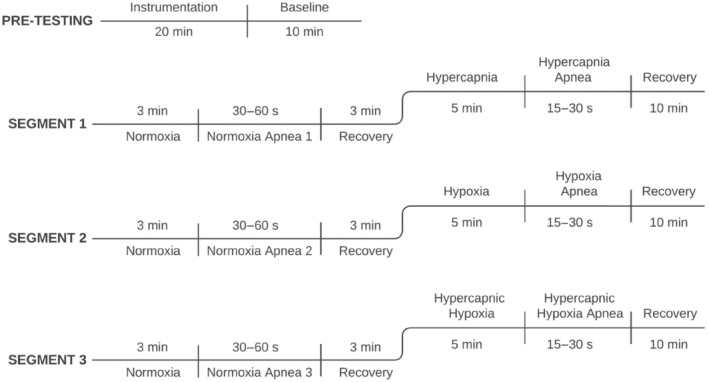
The experimental protocol involved three segments, each with a normocapnic normoxic apnea (end‐tidal O_2_ [P_ET_O_2_] and CO_2_ [P_ET_CO_2_] matched to pre‐testing baseline). The first segment included the hypercapnic condition in which participants were exposed to increased CO_2_ such that P_ET_CO_2_ increased by 5 torr. Segment 1 always occurred first, but segments 2 and 3 were randomized and counterbalanced between participants, with participants blinded to all three gas conditions. The hypoxic condition targeted a peripheral oxygen saturation of 80%–85%, corresponding to approximately 50 torr P_ET_O_2_. The hypercapnic hypoxic condition included a concurrent increase in P_ET_CO_2_ (+5 torr) and a decrease in oxygen saturation (80%–85%). Twenty‐six participants (12 females) completed this standardized protocol.

The study protocol was separated into three segments (Figure 1). Prior to the first segment, a 10‐min baseline period was completed, during which the participant breathed through the mouthpiece to collect a 10‐min average of resting P_ET_O_2_ and P_ET_CO_2_ values. These resting values were used as the baseline for all subsequent gas conditions. Following the initial baseline, the first segment began with normocapnic normoxia via Airforce for 3 min followed by an end‐expiratory apnea to volitional failure. After the apnea and recovery, the participant returned to breathing on Airforce, starting with normocapnic normoxia for 3 min before a 5‐min period of hypercapnia. This 5‐min period has been shown to be sufficient in length to produce an acute ventilatory response to both hypercapnia and hypoxia which is representative of high chemoreflex activation (Steinback & Poulin, 2007). The first segment concluded with a second apnea followed by a 10‐min recovery period. Hypercapnia always occurred in segment 1, but segments 2 and 3 were randomized and counterbalanced between hypoxia and hypercapnic hypoxia. This order was selected because hypoxia, but not hypercapnia, has been shown to elicit a sustained autonomic response that may interfere if applied first (Steinback et al., 2009; Xie et al., 2001). Segments 2 and 3 were identical to segment 1 except hypercapnia was replaced with either hypoxia or hypercapnic hypoxia. Our analysis revealed no order effect between those who underwent hypoxia versus hypercapnic hypoxia first. Participants were blinded to the gas conditions at all times.

### 2.5 Data analysis

All data were collected at 1 kHz and stored for offline analysis using LabChart (ADInstruments, Chart Pro, v8.1.3). Blood pressure waveforms were calibrated to the manual measurements and shifted by −1.25 s to account for both instrument and physiological signal delays. Gas tracings were also shifted by approximately −1.6 s to account for the gas analyzer delay and were subsequently run through a peak detection algorithm to identify end‐tidal values; partial pressures were calculated using current barometric pressure and subtracting water vapor pressure (BTPS). The ECG signal was bandpass filtered (1–50 Hz). Data were extracted beat‐by‐beat into Microsoft Excel for offline analysis (Microsoft Inc., WA, USA). For two participants, we were unable to obtain a clean and reliable ECG signal and instead extracted the data beat‐by‐beat using systolic maxima as a proxy of R–R interval.

Free‐breathing values were calculated as the average for 1 min prior to each apnea. The nadir heart rate was assessed as the longest R–R interval during the final 10 cardiac cycles of each apnea. For any apnea shorter than 10 cardiac cycles (12 total: three during normoxia, three during hypoxia, six during hypercapnic hypoxia) we analyzed the available apnea data and did not supplement the missing data with free‐breathing values prior to apneas. These decisions were consistent for ΔHR and ΔMAP (both discussed below), and Figure 3. The change in heart rate (ΔHR) was calculated as nadir HR minus free‐breathing HR for each respective apnea. Lastly, we compared the HR response to apnea with hypercapnic hypoxia to the summation of individual hypercapnia and hypoxia (hypercapnia+hypoxia). To compare these responses we removed the proportion of the bradycardic response not attributable to each gas by subtracting the normocapnic normoxic ΔHR from each of hypercapnia, hypoxia, and hypercapnic hypoxia such that a ΔHR equivalent to normocapnic normoxia would equal 0 and any greater bradycardia would be a negative number.

Based on the results of our a priori analyses, we completed an additional ad hoc linear regression for each of hypercapnia, hypoxia, and hypercapnic hypoxia to determine the relationship between the ventilatory response to each gas stimulus and the subsequent HR response to their respective apneas. To do so, we used the isolated ΔHR from hypercapnia, hypoxia, and hypercapnic hypoxia without the influence of normocapnic normoxia, as described in the previous paragraph. We completed a similar transformation for our ventilatory data, subtracting the normocapnic normoxic minute ventilation from each gas condition such that a minute ventilation equivalent to normocapnic normoxia would equal 0 and any greater ventilation would be a positive number.

The nadir SpO_2_ was taken during the apnea or within 1 min following the termination of the apnea to account for the circulatory delay in detecting peripheral oxygenation; the change in SpO_2_ (ΔSpO_2_) was calculated as nadir SpO_2_ minus free‐breathing SpO_2_. Apnea duration was determined using the airflow readings from the pneumotach at the start of apnea and the chest movement measured by the respiratory belt at the end of apnea. Mean arterial pressure (MAP) was calculated as diastolic blood pressure + ⅓ pulse pressure. The change in blood pressure (ΔMAP) was calculated by subtracting the resting MAP from the peak MAP occurring during the final 10 cardiac cycles of each apnea. Ventilatory responses were calculated by integrating the sum of the expiratory flow from the pneumotach trace over the minute prior to each apnea to obtain resting minute ventilation.

To identify arrhythmias, a trained member of the research team (BRO) visually scanned ECG tracings during all apneas, identifying any abnormalities in the tracing. All abnormal rhythms were reviewed by a cardiologist (SvD) blinded to study conditions who verified the types and incidence of arrhythmias. Comparisons were then made between the number and types of arrhythmias in apneas completed during the different gas conditions.

All data were first analyzed to identify any possible sex differences; we reported data stratified by sex in Table 1 for transparency. However, no sex differences were observed for our primary outcomes and data were merged for final analyses.

**TABLE 1:** Participant demographics and sex differences.

	All (*n* = 25)	Males (*n* = 13)	Females (*n* = 12)	*p* value
Age (years)	23 ± 2	23 ± 2	23 ± 3	0.520
Height (cm)	172 ± 9	178 ± 8	165 ± 5	<0.001
Weight (kg)	70 ± 14	76 ± 14	65 ± 11	0.042
BMI (kg/m^2^)	24 ± 3	24 ± 3	24 ± 4	0.961
Race	18 White, 4 East Asian, 2 South Asian, 1 mixed White/Asian	8 White, 3 East Asian, 1 South Asian, 1 mixed White/Asian	10 White, 1 East Asian, 1 South Asian	
HR (bpm)	70 ± 9	65 ± 10	75 ± 4	0.005
MAP (mmHg)	88 ± 5	87 ± 4	88 ± 6	0.905
VE (L/min)	13 ± 4	14 ± 5	13 ± 2	0.423
SpO_2_ (%)	98 ± 1	98 ± 1	99 ± 1	0.027
Apnea duration (s)	23 ± 8	25 ± 6	20 ± 8	0.163
Normocapnic normoxia apnea ΔHR (bpm)	−11 ± 15	−13 ± 15	−8 ± 15	0.447

*Note*: All data are reported as mean ± SD.

Abbreviations: HR, heart rate; MAP, mean arterial pressure; SpO_2_, peripheral oxygen saturation; VE, minute ventilation; ΔHR, change in heart rate between free breathing and nadir during apnea.

### 2.6 Statistical analysis

Statistical analyses were performed using Prism 9.0. Data were assessed for normality using histograms and boxplots. We also completed residual analyses prior to the linear regressions. Unpaired *t*‐tests were used to compare male and female data and to compare the beat‐by‐beat heart rate responses to the final 10 cardiac cycles of each apnea between the gas interventions and normocapnic normoxia. To correct for multiple comparisons (*c*), the a priori *⍺* (0.05) was adjusted (*⍺*′) using the experimentwise error rate (*⍺*_e_):

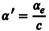

$${⍺}_{e}=1-{\left(1-⍺\right)}^{c}.$$

Continuous variables were analyzed using one‐way repeated measures ANOVA. Holm‐Sidak post‐hoc analyses were performed when the main effects revealed statistical significance. Fisher's exact test was used to compare the number of participants that developed arrhythmias across all of the apneas. Statistical significance was set at *p* < 0.05 for all measurements. Cohen's *d* was used to indicate effect sizes, with 0.2, 0.5, and 0.8 corresponding to small, medium, and large effect sizes, respectively (Cohen, 1992). All data are reported as mean ± SD.

## 3 RESULTS

We successfully tested 12 females and 14 males (23 ± 2 years, BMI 24 ± 3 kg/m^2^). One participant was removed from our final analysis due to large beat‐by‐beat fluctuations in heart rate induced by premature atrial contractions during free breathing and apneas. Sensitivity analysis revealed that this participant's data did not affect our results or interpretations. As such, our final analysis includes 12 females and 13 males. Participant characteristics are reported in Table 1. Males were taller and weighed more than females but cardiovascular parameters were comparable between groups. Further, responses to apneas were the same between sexes.

### 3.1 Responses to gases during free‐breathing

Free‐breathing parameters were comparable between all three normocapnic normoxia conditions. Apnea duration, P_ET_O_2_, MAP, ΔMAP, minute ventilation, and SpO_2_ nadir were also consistent between normocapnic normoxia conditions. Although resting SpO_2_ was statistically lower in the first normocapnic normoxia baseline (mean SpO_2_ = 98 vs. 99 and 99%, *p* < 0.05, *d* = 0.545), and P_ET_CO_2_ was statistically different between normocapnic normoxia preceding hypercapnia and hypercapnic hypoxia conditions (40.3 vs. 41.1 torr, respectively; *p* < 0.05, *d* = 0.268), these differences had small to moderate effect sizes and were likely not physiologically relevant. Resting HR was also statistically higher in the first normocapnic normoxia baseline (mean HR = 70 vs. 66 and 65 bpm, *p* < 0.05, *d* = 0.377), though ΔHR remained similar between all normocapnic normoxia conditions. Free‐breathing cardiovascular and ventilatory parameters are reported in Table 2. HR was higher in all three gas conditions (hypercapnia = 73 ± 10 bpm, hypoxia = 83 ± 12 bpm, hypercapnic hypoxia = 86 ± 12 bpm) compared to normocapnic normoxia (normocapnic normoxia = 70 ± 9 bpm; all *p* < 0.001; *d* = 0.310, *d* = 1.25, *d* = 1.51 respectively). MAP was also higher in all three gas conditions compared to normocapnic normoxia (normocapnic normoxia = 88 ± 5 mmHg) with the largest increase in hypercapnic hypoxia (Table 2). Minute ventilation was higher in all three gas conditions (hypercapnia = 22 ± 7 L/min, hypoxia = 26 ± 9 L/min, hypercapnic hypoxia = 38 ± 10 L/min) compared to normocapnic normoxia (normocapnic normoxia = 13 ± 4 L/min; all *p* < 0.001; *d* = 1.58, *d* = 1.87, *d* = 3.28 respectively).

**TABLE 2:** Free breathing cardiovascular and ventilatory parameters prior to apnea (*n* = 25, 12 females).

	Normocapnic normoxia	Hypercapnia	Hypoxia	Hypercapnic hypoxia
HR (bpm)	70 ± 9	73 ± 10*	83 ± 12* ^,^ **	86 ± 12* ^,^ ** ^,^ ***
MAP (mmHg)	88 ± 5	92 ± 6*	94 ± 9*	96 ± 10* ^,^ **
SBP (mmHg)	115 ± 7	121 ± 10*	123 ± 12*	126 ± 13* ^,^ **
DBP (mmHg)	74 ± 6	77 ± 6*	79 ± 9*	81 ± 10*
VE (L/min)	13 ± 4	22 ± 7*	26 ± 9* ^,^ **	38 ± 10* ^,^ ** ^,^ ***
SpO_2_ (%)	98 ± 1	98 ± 1*	86 ± 3* ^,^ **	83 ± 4* ^,^ ** ^,^ ***
P_ET_O_2_ (mmHg)	91.3 ± 5.1	89.9 ± 5.4*	48.9 ± 1.0* ^,^ **	48.6 ± 2.2* ^,^ **
P_ET_CO_2_ (mmHg)	40.3 ± 3.2	46.2 ± 3.5*	40.6 ± 3.1**	46.3 ± 2.5* ^,^ ***

*Note*: All data are reported as mean ± SD.

Abbreviations: DBP, diastolic blood pressure; HR, heart rate; MAP, mean arterial pressure; P_ET_CO_2_, end‐tidal partial pressure of carbon dioxide; P_ET_O_2_, end‐tidal partial pressure of oxygen; SBP, systolic blood pressure; SpO_2_, peripheral oxygen saturation; VE, minute ventilation.

* *p* < 0.05 versus normocapnic normoxia.

** *p* < 0.05 versus hypercapnia.

*** *p* < 0.05 versus hypoxia.

### 3.2 Responses to voluntary apnea compared to free‐breathing

We observed consistent responses to apneas that align with previous findings. After normocapnic normoxia, apneas caused a large decrease in heart rate (*p* = 0.001, *d* = 0.88). We also observed consistent bradycardia from apneas with hypercapnia (*p* < 0.001, *d* = 1.12), hypoxia (*p* < 0.001, *d* = 1.35), and hypercapnic hypoxia (*p* < 0.001, *d* = 1.29).

### 3.3 Responses to voluntary apnea after normocapnic normoxia

All apnea data are presented in Table 3. Apneas after normocapnic normoxia induced a modest bradycardic response and a concomitant increase in MAP (−11 ± 15 bpm and +14 ± 7 mmHg, respectively). We also observed a small decrease in SpO_2_ (−2 ± 2%). Participants were instructed to complete all apneas to volitional failure, and they held their breath for 23 ± 8 s.

**TABLE 3:** Cardiovascular responses to apneas (*n* = 25, 12 females).

	Normocapnic normoxia	Hypercapnia	Hypoxia	Hypercapnic hypoxia
HR nadir (bpm)	59 ± 16	59 ± 15	64 ± 18	67 ± 18* ^,^ **
ΔHR (bpm)	−11 ± 15	−14 ± 14	−19 ± 15 * ^,^ **	−19 ± 16* ^,^ **
MAP peak (mmHg)	101 ± 10	106 ± 11	105 ± 15	105 ± 15
ΔMAP (mmHg)	+14 ± 7	+14 ± 8	+12 ± 9	+10 ± 9**
SpO_2_ nadir (%)	96 ± 2	97 ± 2*	84 ± 5* ^,^ **	80 ± 8* ^,^ ** ^,^ ***
ΔSpO_2_ (%)	−2 ± 2	−1 ± 2	−1 ± 4	−3 ± 6
Apnea duration (s)	23 ± 8	19 ± 7*	13 ± 4* ^,^ **	10 ± 4* ^,^ ** ^,^ ***

*Note*: All data are reported as mean ± SD.

Abbreviations: HR, heart rate; MAP, mean arterial pressure; SpO_2_, peripheral oxygen saturation; ΔHR, change in heart rate between free breathing and nadir during apnea; ΔMAP, change in mean arterial pressure between free breathing and peak during apnea; ΔSpO_2_, change in peripheral oxygen saturation between free breathing and nadir during apnea.

* *p* < 0.05 versus normocapnic normoxia.

** *p* < 0.05 versus hypercapnia.

*** *p* < 0.05 versus hypoxia.

### 3.4 Responses to voluntary apnea after hypercapnia

Apnea duration was shorter during hypercapnia (19 ± 7 s) than normocapnic normoxia. Voluntary apnea after hypercapnia elicited bradycardia that was similar to normocapnic normoxia (−14 ± 14 bpm, *p* = 0.134 vs. normocapnic normoxia, *d* = 0.204). Further, MAP increased and SpO_2_ decreased to a similar extent between hypercapnia and normocapnic normoxia.

### 3.5 Responses to voluntary apnea after hypoxia

Voluntary apnea after hypoxia elicited greater bradycardia than normocapnic normoxia (−19 ± 15 bpm, *p* = 0.002 vs. normocapnic normoxia, *d* = 0.569) and hypercapnia (*p* = 0.012, *d* = 0.392). Apnea duration was also shorter with hypoxia (13 ± 4 s) compared to both hypercapnia and normocapnic normoxia. However, MAP increased and SpO_2_ decreased to a similar extent between hypoxia and normocapnic normoxia.

### 3.6 Responses to voluntary apnea after hypercapnic hypoxia

Voluntary apnea after hypercapnic hypoxia elicited a greater bradycardia than normocapnic normoxia (−19 ± 16 bpm, *p* = 0.002 vs. normocapnic normoxia, *d* = 0.550). The HR response to hypercapnic hypoxia approached statistical significance compared to hypercapnia (*p* = 0.059, *d* = 0.378) but was comparable to hypoxia (*p* = 0.999, *d* = 0). Apnea duration was shortest during hypercapnic hypoxia (10 ± 4 s). MAP increased and SpO_2_ decreased to a similar degree compared to normocapnic normoxia.

### 3.7 Response to hypercapnic hypoxia versus hypercapnia + hypoxia

**FIGURE 2:**
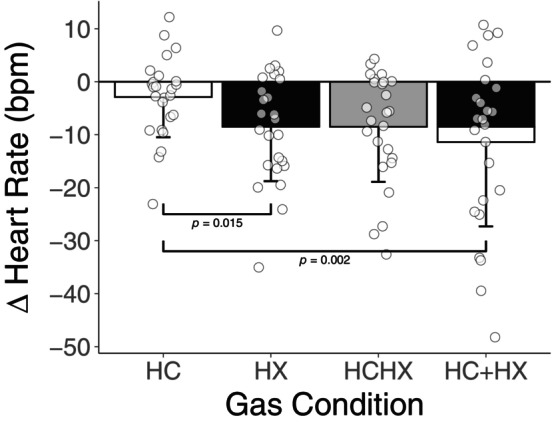
Isolated heart rate responses to apnea for hypercapnia (HC), hypoxia (HX), hypercapnic hypoxia (HCHX), and the summation of hypercapnia and hypoxia (HC + HX; the shading depicts the relative contributions of hypercapnia and hypoxia in the summation; all *n* = 25, 12 females). The *y*‐axis depicts the difference in heart rate from free breathing to nadir during apnea after removing the change in heart rate attributed to normocapnic normoxia (a negative value indicates a greater bradycardic response during gas condition). Statistically significant pairwise comparisons are presented in the figure. All comparisons without *p* values are not statistically significant (*p* > 0.05).

After the removal of the change in heart rate induced by normocapnic normoxia, there was no statistically significant difference between the summation of the bradycardic response to hypercapnia + hypoxia (ΔHR = −9 ± 10 bpm) versus the response to hypercapnic hypoxia (ΔHR = −11 ± 16 bpm, *p* = 0.485, *d* = 0.215; Figure 2).

### 3.8 Arrhythmias

There was a higher prevalence of arrhythmias in apneas during hypercapnic hypoxia than during hypoxia, which in turn had more than during hypercapnia and normocapnic normoxia which both had an equivalent prevalence (Table 4). There was no statistically significant difference between the number of participants who had arrhythmias during apneas after normocapnic normoxia (*n* = 4), hypercapnia (*n* = 3), hypoxia (*n* = 4), or hypercapnic hypoxia (*n* = 7; *p* = 0.628). Hypercapnic hypoxia had the highest number of premature atrial contractions and sinus pauses while hypoxia produced the most first‐degree atrioventricular (AV) blocks. All arrhythmias were supraventricular, originating from the atria or the conduction between the atria and ventricles. Table S1 (https://figshare.com/s/c25dd03edb6530aae6f0) shows that two participants experienced arrhythmias in all six apneas, while six other participants developed arrhythmias in only some of the six apneas. This highlights a low incidence of arrhythmias amongst participants as only 8 out of 26 exhibited arrhythmias throughout the protocol.

**TABLE 4:** Number of arrhythmias in each of the six maximal apneas (*n* = 26, 12 females).

	NX apnea 1	NX apnea 2	NX apnea 3	HC apnea	HX apnea	HCHX apnea
Ectopy						
Premature atrial contractions	1	1	1	1	2	3
Ectopic atrial beats/rhythm				1	1	
Atrial escape beats following sinus pause					1	1
Junctional escape beats following sinus pause	1	1	1	1		
SA or AV node dysfunction						
1st degree AV block			1		2	1
Sinus pause	1	1	1	1		4
Other						
ST depression	1	1	1	1	1	1
Long QT interval	1	1	1	1	1	1
Total	5	5	6	6	8	11

*Note*: Classification of arrhythmias based on type of event. The number of arrhythmias is listed for the three normocapnic normoxic (NX) apneas, as well as the hypercapnic (HC), hypoxic (HX), and hypercapnic hypoxic (HCHX) apneas across all participants. All cardiac events are listed here, including when multiple events were present in a single apnea. The majority of participants did not develop arrhythmias.

## 4 DISCUSSION

### 4.1 Primary findings

**FIGURE 3:**
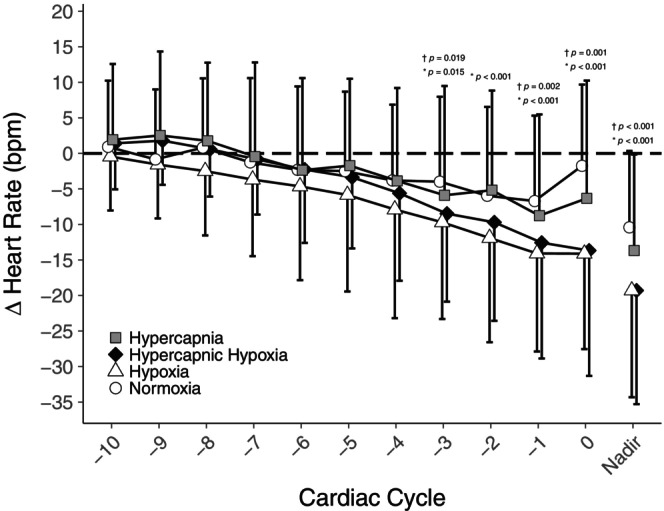
Mean (± SD) change in heart rate during the final 10 cardiac cycles of apnea after normocapnic normoxia (circles), hypercapnia (squares), hypoxia (triangles), and hypercapnic hypoxia (diamonds) relative to the resting heart rate averaged from 1 min preceding each respective apnea (*n* = 25, 12 females). The nadir is the mean response of each participant's single lowest beat during the final 10 cardiac cycles of each apnea. *comparison between normocapnic normoxia versus hypoxia; †comparison between normocapnic normoxia versus hypercapnic hypoxia.

We aimed to compare the cardiovascular responses to apneas during hypoxia, hypercapnia, and combined hypercapnic hypoxia. Our findings demonstrate that unlike hypoxia, hypercapnia (P_ET_CO_2_ +5.8 ± 1.0 torr) does not appear to enhance apnea‐induced bradycardia compared to the normocapnic normoxic condition. Additionally, the bradycardic response to combined hypercapnic hypoxia was similar to that during hypoxia and not further augmented as we had hypothesized (Figure 3). Together these findings indicate that hypoxia elicits a unique parasympathetic augmentation during apnea.

### 4.2 Physiological mechanisms

During short‐term exposure to hypoxia, peripheral chemoreflex activation is heightened. Chemoreflex signaling through the brainstem increases cardiac vagal outflow which is unmasked with the cessation of breathing during an apnea (Busch et al., 2021). This signaling process results in augmented bradycardia compared to apneas performed after normocapnic normoxia because of the added chemoreflex stimulus on top of the universally observed increase in cardiovagal outflow. Our results align with previous findings of this heightened bradycardia during hypoxic apneas (Busch et al., 2021). However, contrary to our hypothesis we did not observe a similarly augmented bradycardia during apneas performed during hypercapnia. This is somewhat surprising given other responses commonly measured during hypercapnic exposure, namely hyperventilation and elevated sympathetic outflow. The deviation in the efferent cardiac response between hypercapnic and hypoxic apneas has several potential origins: differences in afferent chemoreflex signaling, differences in the central processing of the autonomic inputs, differences in cardiac conductivity, or other possible confounding effects. The peripheral chemoreflex is known to respond to hypoxemia below 60 torr (Biscoe et al., 1970), and given that our hypoxic stimulus was 50 torr, the peripheral chemoreflex is undoubtedly activated. One possible explanation is that our hypercapnic stimulus was inadequate to activate the chemoreflexes to the same extent as hypoxia. However, hypercapnia induced a substantial ventilatory response, indicating that a stimulus was present. Further, the ventilatory responses to hypercapnia and hypoxia were comparable, which suggests similar chemoreflex activation between conditions. However, ventilatory responses may not correlate with peripheral sympathetic nerve activity (Keir et al., 2019) and it is unclear if chemoreflex activity correlates to efferent cardiac vagal outflow.

Differences in cardiac responses to hypercapnia and hypoxia may also arise from differing central processing. Central chemoreceptors are dispersed throughout the brainstem in regions such as the retrotrapezoid nucleus (Guyenet, 2014). This creates a multitude of connections between central chemoreceptors and adjacent brainstem nuclei, many of which could further influence cardiac vagal nuclei in the brainstem. Compared to the peripheral chemoreflex, which connects directly from the periphery to the nucleus of the solitary tract (Dampney, 2016; Takakura et al., 2006), the divergence in responses between hypercapnia and hypoxia may originate in central hypercapnic signaling. Another factor to consider in central hypercapnic signaling is glial cells, which have been shown to be chemosensitive, responding to changes in CO_2_ and mediating the hypercapnic response during free‐breathing (Huckstepp & Dale, 2011). Future research using functional imaging techniques or animal models should be performed to investigate differences in brain signaling between hypoxic and hypercapnic apneas.

The difference in the HR response to hypercapnic and hypoxic apneas could also be caused by variations in how the two stimuli affect cardiac conduction. Hypoxia has been shown to decrease cardiac contractility gradually as oxygen saturation decreases (Walley et al., 1988), and hypercapnia has shown a similar capacity to affect contractility (Cingolani et al., 1990). The ability to affect contractility demonstrates that both hypoxia and hypercapnia can directly influence heart tissue. However, not all heart tissue is equally affected by these stimuli; pacemaker cells in the sinoatrial (SA) node are much more resilient than cardiac myocyte cells (Kohlhardt et al., 1977). Using animal models, the SA and AV nodes have been shown to withstand prolonged hypoxic stimuli because of an abundance of energy produced through the anaerobic glycolytic pathway (Nishi et al., 1980; Senges et al., 1979). Further research using cellular models of induced pacemaker cells indicates that these cells have low metabolic demands and are highly resistant to various stressors (Gu et al., 2019). To our knowledge, it is unknown whether hypercapnia and hypoxia differentially affect cardiac conduction in humans.

Finally, there are multiple confounding factors external to the chemoreflex response that may influence our results and interpretations. The baroreflex can affect HR responses if blood pressure changes to a different extent between conditions (Di Rienzo et al., 2009). However, we observed similar increases in MAP between conditions and further noticed no differences in peak MAP. Although ΔMAP was greater in hypercapnia than hypercapnic hyperoxia, this difference would logically cause greater baroreflex‐induced bradycardia in the former condition which is the opposite of the observed effect. Apnea duration may also affect the degree of bradycardia (Engan et al., 2013). Our participants were instructed to hold their breath until volitional failure, and apneas after hypoxia had much greater bradycardia than hypercapnia despite their shorter duration, indicating that time should not have been a limiting factor in and of itself.

During the combined hypercapnic hypoxia condition, we observed no additional bradycardia versus the hypoxia condition (Figure 2). The lack of potentiation further reinforces that hypercapnia does not appear to contribute to apnea‐induced bradycardia, either directly (by activating the central or peripheral chemoreceptors) or indirectly (by increasing the sensitivity of the peripheral chemoreceptors to O_2_). While a floor effect of HR may explain our results, such that no further reflex bradycardia may be elicited beyond that observed during hypoxia, the absolute nadir HR during hypercapnic hypoxia apneas was 8 bpm higher than that seen during normocapnic normoxia, suggesting this is unlikely. These data indicate that the bradycardic response to apneas with hypercapnic hypoxia is only due to a hypoxia‐mediated enhancement of cardiac vagal outflow. Conversely, hypercapnia does not contribute to this response despite clear chemoreflex activation during free breathing. Our results also reaffirm the powerful modulatory role of hypoxia on the strength of the diving response (Busch et al., 2021).

### 4.3 Ventilatory response

**FIGURE 4:**
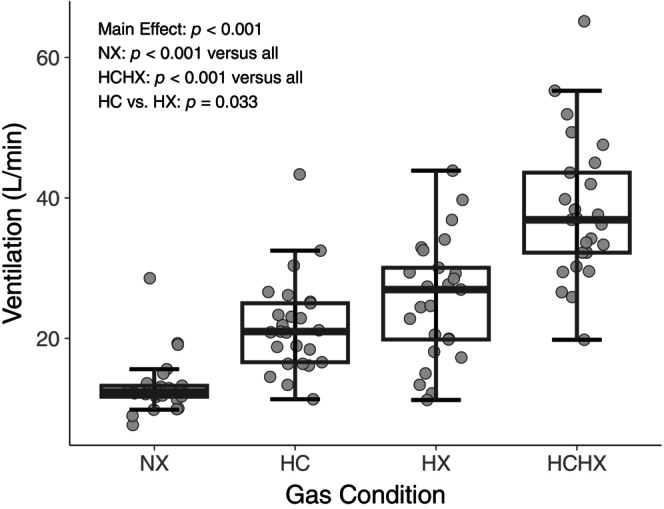
Ventilatory response to normocapnic normoxia (NX), hypercapnia (HC), hypoxia (HX), and hypercapnic hypoxia (HCHX; all *n* = 25, 12 females). ANOVA and post hoc *p* values are presented in the figure. All pairwise comparisons were statistically significant (*p* < 0.05).

**FIGURE 5:**
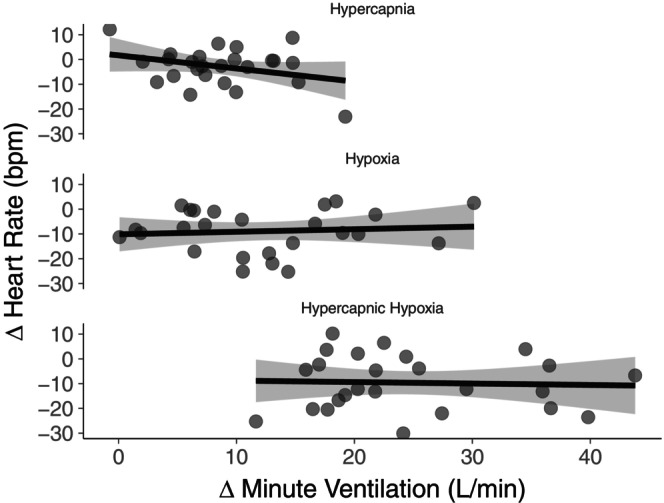
Relationship between the ventilatory response to each gas condition and the subsequent heart rate response to apnea. Linear regression model suggests there is no relationship (hypercapnia: *slope* = −0.198, *p* = 0.114; hypoxia: *slope* = 0.067, *p* = 0.700; hypercapnic hypoxia: *slope* = −0.026, *p* = 0.873; all *n* = 25, 12 females). The *y*‐axis depicts the difference in the change in heart rate from free breathing to nadir during apnea between each respective gas and normocapnic normoxia (a negative value indicates a greater bradycardic response during gas condition). The *x*‐axis depicts the difference in ventilation between each respective gas and normocapnic normoxia (a positive value indicates a greater ventilatory response during gas condition). Shaded area represents the 95% CI of the regression curve.

During free‐breathing, hypercapnia, and hypoxia increased minute ventilation to a similar extent relative to normocapnic normoxia. Further, the ventilatory response to hypercapnic hypoxia was statistically larger than hypercapnia or hypoxia on their own, demonstrating a potentiated ventilatory chemoreflex activation (Figure 4). These ventilatory responses contrast the HR responses, suggesting a divergence in signaling between chemoreflex‐mediated cardiac and respiratory pathways. Based on these findings, we completed an ad hoc linear regression between the ventilatory response to each gas condition and the subsequent bradycardia to their respective apneas (Figure 5). This analysis revealed no relationship between the strength of the preceding ventilatory response and the degree of subsequent bradycardia, aligning with the notion of diverging respiratory and cardiac pathways (Keir et al., 2019).

### 4.4 Secondary outcomes

The duration of apneas indicates an additive effect of hypercapnia and hypoxia. Hypercapnic hypoxia caused the shortest apneas, indicating a heightened drive to breathe due to the combined stimulus of hypercapnia and hypoxia pre‐apnea. Hypoxic apneas were also shorter than hypercapnic apneas on average, indicating that hypoxic chemoreflex activation may have caused a greater drive to breathe than hypercapnia. However, it is also possible that the potency of hypercapnia was lesser than hypoxia based on previous indications that arterial CO_2_ levels drive the urge to breathe during apneas (Pernett et al., 2023; Schagatay et al., 2000).

Our results showed no statistically significant increase in arrhythmias in any gas condition compared to normocapnic normoxia, but we did see the highest prevalence of arrhythmias in the hypercapnic hypoxia condition. This suggests that the increased stress of a combined hypercapnic hypoxic stimulus may cause a higher incidence of arrhythmias, though we had insufficient statistical power to confirm this relationship with greater certainty. These results support previous work showing that short‐term hypoxia does not promote arrhythmias during apneas (Busch et al., 2021), and extends these findings to apneas following short‐term hypercapnia. However, there may be an interaction between acute hypoxia and hypercapnia on arrhythmia incidence.

### 4.5 Considerations

We observed no sex differences in response to apneas in any of the gas conditions. Previous work has shown potential sex differences in the baroreflex response to apneas (Patel et al., 2014) and differing responses to hypercapnic hypoxia during free breathing (Jacob et al., 2020), as well as differing sympathetic nerve activity responses to central chemoreceptor stimulation (Sayegh et al., 2022). However, none of these previous observations have manifested in the cardiac apnea response we have demonstrated.

We observed a high level of variability in heart rate responses between participants. Whereas some participants had large ventilatory or cardiovascular responses, others experienced minimal effects of hypercapnia or hypoxia; others still had strong HR or ventilatory responses but were non‐responders for the other variable. Participants had higher than expected resting minute ventilation during normocapnic normoxia (13.2 ± 4.2 L/min), likely caused by the novelty of the mouthpiece and associated deadspace. However, our Airforce gas system maintained basal end‐tidal values to mitigate the effects of hyperventilation. Further, hyperventilation before apneas does not impact the apnea‐induced bradycardia and, therefore, likely did not impact the results (Pernett et al., 2023). We did not control for the ovarian cycle in females. However, this decision provided greater external validity of our results. Our sample was also recruited using convenience sampling from the university community and may therefore be of higher socioeconomic status and education level than the average population.

### 4.6 Perspectives and significance

The physiology discussed herein has implications beyond the laboratory setting. Professional free‐divers train to withstand superimposed hypercapnia and hypoxia induced by extended underwater breath‐holds (Heusser et al., 2009). Additionally, individuals who sojourn to or live at high altitudes experience chronic hypoxia which can induce substantial bradycardia and a high prevalence of cardiac arrhythmias during voluntary apneas (Busch et al., 2018). Beyond extreme environments, obstructive sleep apnea (OSA) is characterized by intermittent periods of hypercapnia and hypoxia throughout the night resulting from apneas and hypopneas (May et al., 2017). We examined the individual contributions of hypercapnia and hypoxia to the cardiac response to voluntary apneas with hypercapnic hypoxia. Our findings may point to the possibility that nocturnal bradycardia in OSA (Zwillich et al., 1982) largely originates from hypoxic stress, but our young and healthy sample, in addition to our daytime voluntary apneas, limit further extrapolation to clinical populations.

In summary, we demonstrated that hypercapnia does not influence bradycardia during apneas. Furthermore, the bradycardic response to apneas after hypercapnic hypoxia is only influenced by hypoxia and not further enhanced by hypercapnia. Overall, under apneic conditions, hypoxia rather than hypercapnia drives the cardiac component of the diving response.

## AUTHOR CONTRIBUTIONS

B.R.O. contributed to the design of the study, participant recruitment and data collection, data analysis, assessment of the electrocardiogram trace for abnormalities, statistical analysis, and writing the manuscript. D.A.Y. contributed to the design of the study, participant recruitment and data collection, data analysis, statistical analysis, and writing the manuscript. L.E.M. contributed to data collection. S.v.D. contributed to data analysis including identification and classification of arrhythmias. T.A.D. contributed to the conceptualization and design of the study. C.D.S. contributed to the conceptualization and design of the study. All authors reviewed and approved the final version of the manuscript. All authors agree to be accountable for all aspects of the work in ensuring that questions related to the accuracy or integrity of any part of the work are appropriately investigated and resolved. All persons designated as authors qualify for authorship, and all those who qualify for authorship are listed.

## FUNDING INFORMATION

Craig Steinback NSERC Discovery Grant RGPIN‐2020‐05385. Benjamin O'Croinin NSERC CGS‐M.

## CONFLICT OF INTEREST STATEMENT

The authors disclose no conflicts of interest.

## ETHICS STATEMENT

This study was approved by the University of Alberta (Pro00096895) and Mount Royal University (Protocol #103970) Health Research Ethics Boards. The study conformed with the latest version of the *Declaration of Helsinki* except for registration in a public database.

## Supporting information


Table S1:

Table S2:

## Data Availability

Raw data are available from the corresponding author upon reasonable request.
