# Supplementary material for: Influence of hypercapnia and hypercapnic hypoxia on the heart rate response to apnea
Source: Physiol Rep. 2024 Jun 14;12(11):e16054. doi: 10.14814/phy2.16054 (PMC11176737; doi:10.14814/phy2.16054)
Supplement: Supplementary file 1 — Table S1: [file PHY2-12-e16054-s001.docx]

| Supplementary Table 1. Arrhythmias seen during the six maximal apneas in the eight participants with arrhythmogenesis. | | | | | | |
| --- | --- | --- | --- | --- | --- | --- |
| Participant | Normocapnic Normoxic Apnea 1 | Normocapnic Normoxic Apnea 2 | Normocapnic Normoxic Apnea 3 | Hypercapnic Apnea | Hypoxic Apnea | Hypercapnic Hypoxic Apnea |
| 1 | Premature atrial contraction | Premature atrial contractions | Premature atrial contractions | Premature atrial contractions; ectopic atrial beats | Premature atrial contractions; first degree AV block; ectopic atrial beats | Premature atrial contractions |
| 2 |  |  |  |  | Premature atrial contractions with atrial escape beats | Sinus pause with atrial escape beats; premature atrial contractions |
| 3 | Sinus pause; junctional escape beat | Sinus pause; junctional escape beat | Sinus pause; junctional escape beat | Sinus pause; junctional escape beat |  |  |
| 4 |  |  |  |  |  | Sinus pause; premature atrial contraction |
| 5 |  |  |  |  |  | Sinus pause |
| 6 | ST depression; long QT interval | ST depression; long QT interval | ST depression; long QT interval | ST depression; long QT interval | ST depression; long QT interval | ST depression; long QT interval |
| 7 |  |  | First degree AV block |  | First degree AV block | First degree AV block |
| 8 |  |  |  |  |  | Sinus pause |
| Classification of the arrhythmias for all participants that exhibited cardiac events during apneas. 8 participants (30%) developed arrhythmias, with participants 1 and 6 having cardiac events in all apneas. Multiple arrhythmias occurred during some apneas. | | | | | | |
