# Supplementary material for: Influence of hypercapnia and hypercapnic hypoxia on the heart rate response to apnea
Source: Physiol Rep. 2024 Jun 14;12(11):e16054. doi: 10.14814/phy2.16054 (PMC11176737; doi:10.14814/phy2.16054)
Supplement: Supplementary file 2 — Table S2: [file PHY2-12-e16054-s002.docx]

| Supplementary Table 2. Ovarian cycles and contraceptive use for all female participants (n=12). | | |
| --- | --- | --- |
| **Participant** | **Contraceptive** | **Days Since Last Menses*** |
| A | Intrauterine device | Not menstruating |
| B | None | 26 |
| C | Intrauterine device | 10 |
| D | Intrauterine device | 18 |
| E | Oral contraceptive (pill phase on day of testing) | 22 |
| F | Intrauterine device | 6 |
| G | None | 20 |
| H | None | 25 |
| I | None | 19 |
| J | Intrauterine device | Not menstruating |
| K | Oral contraceptive (placebo phase on day of testing) | 2 |
| L | None | 25 |
| *Note: integer value indicates number of days between the first day of menses of the most recent ovarian cycle and the day of testing, as self-reported by female participants. | | |
